# Physicochemical property, bacterial diversity, and volatile profile during ripening of naturally fermented dry mutton sausage produced from Jianzhou big-eared goat

**DOI:** 10.3389/fmicb.2022.961117

**Published:** 2022-09-02

**Authors:** Juan Chen, Ying Niu, Jie Wang, Ziyao Yang, Zijian Cai, Xiaofang Dao, Chengen Wang, Yong Wang, Yaqiu Lin

**Affiliations:** ^1^College of Food Science and Technology, Southwest Minzu University, Chengdu, China; ^2^Sichuan Tian Di Yang Bioengineering Limited Corporation, Chengdu, China; ^3^College of Animal and Veterinary Sciences, Southwest Minzu University, Chengdu, China

**Keywords:** naturally fermented mutton sausage, physicochemical properties, bacterial community, volatile flavors, correlation between bacteria and volatiles

## Abstract

Physicochemical properties, bacterial communities, and volatile compounds of mutton sausage produced from Jianzhou Big-Eared goat meat during natural ripening were investigated. *Firmicutes* and *Bacteroidetes* accounted for over 66% of all operational taxonomic units (OTUs) throughout the whole process, with *Lachnospiraceae*_NK4A136_group and *Staphylococcus* as the predominant genus during the early and later ripening periods, respectively. The evolution of microbial composition became less rich and diverse. The uncultured bacterium, the *Lachnospiraceae*_NK4A136_group, and *Staphylococcus* were marker bacteria on days 0, 7, and 26, respectively, with none on day 16. The bacteria distribution seemed to influence the volatile profile of mutton sausage throughout processing, with the bacterial composition on day 0 and day 7 clustered separately from day 16 and day 26, and the same pattern for the volatile profile. Meanwhile, the concentration of total volatile fraction significantly increased, and the majority of the volatile compounds were generated during late ripening. Non-anal, hexanal, decanal, heptanal, dodecyl aldehyde, benzaldehyde, 3-methylbutanal, γ-dodecalactone, 2-pentylfuran, and 1-octen-3-ol were key volatile compounds, contributing to the overall mutton sausage flavors. Based on Spearman’s correlation analysis, *Staphylococcus* as well as *Psychrobacter* were positively correlated with the production of the key volatile compounds, and other bacteria such as *Lachnospiraceae_*NK4A136_group, *Bacteroides*, *Lactobacillus*, *Prevotella*_1, *Odoribacter*, and so on were associated with the production of most alcohols and esters.

## Introduction

Although sheep or goat meat was not the most consumed meat in the world from 1994 to 2004, the production of sheep and goats notably increased by 75 and 42%, respectively, with a sustained trend until 2018 (Teixeira et al., 2020). Sheep meat includes many vitamins, minerals, and essential polyunsaturated fatty acids (PUFAs) (Junkuszew et al., 2020). The reduced calorie and fat contents coupled with more riboflavin have been incentives to consume goat meat according to United States Development of Agriculture (USDA) Nutrient Database reports (Bratcher et al., 2011). Jianzhou Big-eared goat is the second species of meat goat following the Nanjiang Huang goat in China. Jianzhou Big-Eared goat meat is popular for daily consumption in Sichuan, especially in the winter season, in which it is treated with certain processing steps such as roasting and preparing as mutton soup.

There are some traditionally fermented mutton sausages recognized for their individuality and associated with their consumption history in the regions that produce them. Some of them are fermented, fresh, or smoked sausages. Sucuk is a Turkish-style sausage made of beef, sheep, and goat meat, and tail fat, which is very popular in Turkey and several Middle East countries, as well as Europe (Stajić et al., 2013). Fjellmorr and Lambaspaeipylsa are dried fermented sausages, containing lamb, beef, and pork, that are traditionally produced in Norway and Iceland (Teixeira et al., 2020). Pitina is a typical fermented meat product manufactured from sheep or goat, mixed with pork lard, smoked, dried, and ripened, made in mountainous areas of Northeast Italy since the first half of the XIX century (Iacumin et al., 2020). Geema and Arijia are traditional goat meat produced from Kumaun Himalaya in Northern India (Teixeira et al., 2020). These fermented mutton sausages are produced around the world due to their convenience and peculiar sensory characteristics. They constitute a cultural heritage as seen by the high diversity of products in Europe, Asia, and Africa.

The microbial ecology of naturally fermented sausages is diverse with many genera and species cohabiting (Talon and Leroy, 2011). There was a big difference in the microbiota of traditionally fermented sausages in four typical regions of China (Chengdu, Shenzhen, Changsha, and Harbin); however, lactic acid bacteria and cyanobacteria were dominant overall (Huang et al., 2021). A high difference among Salame Piacentino PDO, a typical dry fermented sausage from northern Italy, produced by six factories in Piacenza was reported, and the main bacterial species involved in its fermentation were confirmed to be *Staphylococci* and *Lactobacilli* dominating in salamis from three factories, respectively (Połka et al., 2015). Lactic acid bacteria (particularly *Lactobacillus*) and coagulase-negative *cocci* (mainly *Staphylococcus*) have been reported as the most important bacterial groups in a high-value Spanish dry fermented sausage traditionally manufactured with the activity of autochthonous microbiota (Quijada et al., 2017). The great diversity of the indigenous microbiota in naturally fermented sausages was linked to the raw materials (raw meats and casings), the environment of manufacturing, and types of equipment (Talon et al., 2007).

The microbial metabolism reaction is one of the important factors influencing the characteristics of naturally fermented meat, through the conversion of carbohydrates, amino acids, and lipids to aroma compounds with various aroma notes (Ravyts et al., 2012; Flores and Piornos, 2021). In recent years, many attempts have been made to correlate bacterial diversity with the formation of volatile aroma compounds in traditionally fermented sausages. For traditional dry sausages from different regions in northeast China, positive correlations based on Spearman’s correlation analysis were found between *Weissella hellenica*, *Lactobacillus sakei*, *Lactococcus lactis*, *Lactobacillus alimentarius*, *Lactobacillus plantarum*, and carboxylic acids and alcohols; *L. lactis*, *L. alimentarius*, and *L. plantarum* were associated with the production of most esters, aldehydes, and ketones (Hu et al., 2020). The indigenous microbiota of the traditional Italian Felino sausages, including *L. lactis*, *Lactobacillus citreum*, *Lactobacillus gelidum*, *Staphylococcus xylosus*, and *L. sakei*, displayed higher counts of KEGG genes involved in *ex novo* fatty acid biosynthesis and amino acid metabolism, leading to a higher production of long chain esters such as ethyl octanoate and decanoate at the end of ripening (Ferrocino et al., 2018).

As to fermented mutton sausage, some pieces of research evaluated the effects of inoculation with different starter cultures on microbiological and physicochemical properties (Zhao et al., 2011; Arief et al., 2014; Wang et al., 2019); however, as far as we know, limited information about the exact composition of the microbial successions and the correlating volatile profile has been reported on spontaneously fermented mutton sausage. Fermented mutton sausage derived from Jianzhou Big-Eared goats has not been reported so far. In this study, by high-throughput sequencing and chromatographic analysis, we performed physicochemical properties, bacterial profiles, and volatile metabolites characterization of mutton sausage from Jianzhou Big-Eared goats during natural fermentation. Correlations between the microflora and volatile compounds of the mutton sausages in different stages were further examined. These results may provide references to investigate the interaction between bacterial communities and the metabolism profile in the natural fermentation ecosystem of mutton sausage.

## Materials and methods

### Mutton sausage preparation and sampling

The production was performed in the Food Science and Engineering Laboratory of Southwest Minzu University and was carried out in the period February–March 2021. Fresh lean mutton and back fat of Jianzhou Big-Eared goat were provided by the Sichuan Tian Di Yang Bioengineering Limited Corporation (Jianyang, Chengdu, Sichuan). The lean mutton (4,000 g) and fat (1,000 g) were minced through a 15.0-mm plate with the following additives (ranges g/kg meat): sucrose (10), salt (25), sodium nitrate (0.07), sodium nitrite (0.07), and Na-L-ascorbate (0.5). The ground meat was thoroughly mixed with additives and maintained for 24 h at 0 to 4°C in the refrigerator. Then, the meat was stuffed into natural pig intestine casings and then hung vertically outdoors, ripening for 26 days spontaneously at a temperature between 10and 25°C, with relative humidity between 45 and 90%.

Two independent batches of mutton sausages with different mutton lean and back fat were prepared at the same time. For different processing stages of each batch, sampling was carried out on day 0 (labeled as Group_S1), day 7 (labeled as Group_S2), day 16 (labeled as Group_S3), and day 26 (labeled as Group_S4). At each sampling time, six observations (triplicate observations for each batch) were collected and subjected to pH, moisture content, nitrite ion concentration, thiobarbituric acid reactive substances (TBARS), color, bacterial population, and microbial diversity analyses, and four observations (duplicate observations for each batch) were collected and subjected to metabolic analyses. In this procedure, two bathes of sausages (replicates) were included as random terms, and ripening time (0, 7, 16, and 26 days) was included as a fixed effect.

### Physicochemical indexes measurement

#### pH, moisture content, nitrite, and thiobarbituric acid reactive substances measurement

The pH was measured with a pH meter (pH-STAR, MATTHAUS, Germany) at room temperature (approximately 25°C). Moisture content was determined using METTLER-TOLEDO HR83.

Nitrite was determined using the spectrophotometry method described by GB 5009.33-2016 (China). In brief, 12.5 mL of 50 g/L saturated borax solution was added to 5 g of each chopped sausage sample, 150 mL of water was added at 70°C, and the sample was heated in a boiling water bath for 15 min. About 5 mL of 106 g/L potassium ferrocyanide solution and 5 mL of 220 g/L zinc acetate solution were added to precipitate protein. After the static stand for 30 min, fat was removed, and then, the filtrate was collected. Then, 40 mL of filtrate, 2 mL of 4 g/L p-aminobenzenesulfonic acid solution, and 1 mL of 2 g/L naphthalene ethylenediamine hydrochloride solution were mixed, and water was added and allowed to stand for 15 min. The absorbance was measured at 538 nm on a spectrophotometer. The sodium nitrite serial standard solutions were prepared and detected. Nitrite concentrations of samples were quantified through an external standard method.

Lipid oxidation was evaluated by the determination of TBARS according to the procedure of O’Keefe and Wang (2006). In brief, before the test, the 6-tetramethoxypropane (TMP) serial standard solutions were prepared. Mutton sausage samples were first homogenized with distilled water, 10% sodium dodecyl sulfate (SDS), and 0.01% propyl gallate and 0.02% EDTA solution. One mL of homogenate and 1.0 mL of each standard solution were, respectively, mixed with 4.0 mL of a mixture solution (0.375% 2-thiobarbituric acid, 0.506% SDS, and 11.7% of 80% acetic acid, final pH of 3.4), heated at 95°C for 60 min, and cooled to room temperature at last. Then, 5 mL of the solution (1:15 ratio of pyridine to n-butanol) was added and mixed. The samples and standard solutions were centrifuged. The organic layer was then collected and measured at 532 nm on a spectrophotometer. The equivalent TMP concentration (μmol/L) of the samples was determined by a standard curve developed from the absorbance of the TMP standard solutions. The final concentration conversion of TMP to malonaldehyde (MDA) was obtained by multiplying the number of μmol/L of TMP equivalent per gram of sample by the molecular weight of MDA. The final unit of TBARS value was expressed as mg of MDA equivalent per kg of mutton sausage (mg MDA equivalent/kg).

#### Color measurement

Color measurements were carried out using a Chroma Meter (CR-400, Konica Minolta Sensing Europe). The chroma meter was standardized with a white Minolta calibration plate. L*(lightness), a*(redness), and b*(yellowness) scale coordinates were obtained for the flat surfaces of ground mutton sausages by averaging three readings.

### Bacterial population measurement

The bacterial counts of mutton sausage samples were determined according to the method of Chen et al. (2019). Total aerobic counts were detected using Plate Count Agar (PCA; Qing Dao Hope Bio-Technology Limited Corporation, Qing Dao City, China) after incubation at 37°C for 48 h. Presumptive lactic acid bacteria counts were determined on de Man Rogosa Sharpe Agar (MRSA, Qing Dao Hope Bio-Technology Limited Corporation, Qing Dao City, China) after incubation at 30°C for 48 h. *Staphylococci* counts were cultured on Mannitol Salt Agar (MSA, Qing Dao Hope Bio-Technology Limited Corporation, Qing Dao City, China) after incubation at 32°C for 17 h.

### Microbial diversity analysis

#### DNA extraction and amplification

Mutton fermented sausage samples were snap frozen and stored at −80°C after collection. Bacterial DNA was isolated from the sausage samples using a DNeasy PowerSoil kit (Qiagen, Hilden, Germany) following the manufacturer’s instructions. DNA concentration and integrity were measured by a NanoDrop 2000 spectrophotometer (Thermo Fisher Scientific, Waltham, MA, United States) and agarose gel electrophoresis, respectively. PCR amplification of the V3–V4 hypervariable regions of the bacterial 16S rRNA gene was carried out in a 25-μL reaction using universal primer pairs (343F: 5′-TACGGRAGGCAGCAG-3′; 798R: 5′-AGGGTATCTAATCCT-3′). The reverse primer contained a sample barcode, and both primers were connected with an illumine sequencing adapter.

#### Library construction and sequencing

The amplicon quality was visualized using gel electrophoresis. The PCR products were purified with Agencourt AMPure XP beads (Beckman Coulter Co., United States) and quantified using a Qubit dsDNA assay kit. The concentrations were then adjusted for sequencing. Sequencing was performed on an Illumina NovaSeq6000 with two paired-end read cycles of 250 bases each (Illumina Inc., San Diego, CA, United States; OE Biotech Company; Shanghai, China).

#### Bioinformatic analysis

Paired-end reads were preprocessed using Trimmomatic software to detect and cutoff ambiguous bases (N). It also cutoff low-quality sequences with an average quality score below 20 using a sliding window trimming approach. After trimming, paired-end reads were assembled using FLASH software. Parameters of assembly were as follows: 10 bp of minimal overlapping, 200 bp of maximum overlapping, and 20% maximum mismatch rate. Sequencing was performed by further denoising as follows: reads with ambiguous, homologous sequences or below 200 bp were abandoned. Reads with 75% of bases above Q20 were retained using QIIME software (version 1.8.0). Then, reads with chimera were detected and removed using VSEARCH. Clean reads were subjected to primer sequence removal and clustering to generate operational taxonomic units (OTUs) using VSEARCH software with a 97% similarity cutoff. The representative read of each OTU was selected using the QIIME package. All representative reads were annotated and blasted against the SILVA database (Version 132) using the RDP classifier (confidence threshold was 70%). The microbial diversity in mutton sausage samples was estimated using the alpha diversity that includes the Chao1, Shannon diversity, and Simpson diversity indices. The Bray–Curtis distance matrix performed by QIIME software was used for Bray–Curtis Principal Coordinates Analysis (PCoA) and phylogenetic tree construction. The 16S rRNA gene amplicon sequencing and analysis were conducted by OE Biotech Co., Ltd. (Shanghai, China).

### Volatile compounds analysis

The headspace solid-phase microextraction/gas chromatograph-mass spectrometry (HS-SPME/GC-MS) analysis was performed using a Thermo Trace ultra DSQ II (Thermo Fisher Scientific). The solid-phase microextraction fiber was 50/30 μm divinylbenzene/carboxen/polydimethylsiloxane. The ground mutton sample (5 g) was transferred to a 20-mL glass vial sealed with PTFE caps, after which each sample was supplemented with 10 μL Cyclohexanone (0.475 μg/μL) as the internal standard (IS). All detection steps, including preheating (80°C, 10 min), extraction (80°C, 30 min), desorption (230°C, 2 min), analyzing, and cleaning (250°C, 10 min), were conducted automatically using a Triplus auto-sampler (Thermo Fisher Scientific, Waltham, MA, United States). Volatile compounds were separated using the TG-WAXMS B column (30 m × 0.25 mm × 0.25 μm). A splitless mode was used. The column was held at 40°C for 3 min and was then raised to 210°C at the rate of 5°C min^–1^, and the final temperature was held for 5 min. The carrier gas was ultra-purified helium (99.999% purity) at a flow rate of 1.0 mL/min. Mass spectrometry was used in the electron ionization mode at an ionization energy of 70 eV. The mass scan range of m/z was set from 50 to 500 amu, and a mass transfer line temperature was 220°C.

Volatile compounds were identified by comparing the experimental mass spectra with a mass spectra library from NIST 11 and by calculating the retention index relative to standard alkanes (C8–C26) and again comparing them with those reported in the database^1^. Compounds were identified when the semblance degree was more than 90%. The volatile compounds were semi-qualified by dividing the peak areas of the compounds by a peak area of the IS, and this ratio was multiplied by the ratio of the initial concentration of the IS compared to the weight of the samples (expressed as μg/kg). ROAV was employed to evaluate the contribution of each compound to the overall fermented mutton sausage aroma. Compounds with ROAV ≥ 1 were important in terms of sensory perception. Compounds with 0.1 ≤ ROAV < 1 were considered to have a role in the overall aroma (Zhang et al., 2020).

### Sensory analysis

The sausages were submitted for sensory evaluation to determine the effect of ripening time on the quality of the product. Appearance, color (the intensity of red), flavor, and overall acceptability were evaluated using a hedonic scale, where 1 denotes dislike extremely, 5 denotes neither like nor dislike, and 9 denotes like extremely. The sensory panel consisted of nine trained post-graduate students, with five women and four men. The sausages were cut into slices of 4 mm thickness and served at room temperature on white plastic dishes. Water and unsalted bread were provided for panelists to rinse and clean their mouths between samples.

## Statistics analysis

All the results were expressed as the means (of six or four observations) ± standard errors at each sampling time. Data were statistically analyzed using the one-way ANOVA procedure of SPSS version software (IBM SPSS Statistics 22). Duncan’s multiple range test was used to determine any significant differences between mean values and evaluations. *P*-values less than 0.05 were considered statistically significant.

Beta diversity analysis and statistically significant differences for microbiota and volatile compounds were both determined using multivariate analysis of variance including principal component analysis (PCA), principal coordinate analysis (PCoA), and linear discriminant analysis coupled with effect size measurements (LEfSe). Spearman’s rank correlation analysis was used to estimate the relationship between predominant bacteria and volatile compounds. These multivariate analyses were conducted using OECloud tools at http://cloud.oebiotech.cn.

## Results and discussion

### Physicochemical properties of mutton sausage during ripening

Physical-chemical properties in fermented mutton sausage during ripening are shown in Table 1. The meat after curing (day 0) showed a pH value of 5.55. The evolution of pH exhibited a slow increase trend, reaching a value of 5.99 after a total of 26 days of fermentation. The variation of pH during the spontaneous fermentation of mutton sausage in the present study was consistent with “Pitina,” a traditional peculiar sausage-like product of northeast Italy, whose pH remained almost stable up to day 6 of drying, then an increase of the pH level was registered in subsequent drying and smoking phases, and finally, pH was 5.84 after 37 days of ripening (Iacumin et al., 2020). Moisture content decreased sharply until day 7, then a gradual decrease was observed until day 26. After fermentation and ripening, the moisture content reduced from 43.62 to 3.48% due to dehydration; therefore, the mutton sausage could be considered as dry fermented sausages based on the loss of water of over 30%.

**TABLE 1:** Physicochemical properties of fermented mutton sausage during natural ripening (means ± standard errors).

Day	0	7	16	26
pH value	5.55 ± 0.02^d^	5.76 ± 0.02^c^	5.87 ± 0.03^b^	5.99 ± 0.02^a^
Moisture content (%)	43.62 ± 2.89^a^	15.95 ± 1.49^b^	6.96 ± 0.49^c^	3.48 ± 1.42^c^
Nitrite ion concentration (mg/kg)	29.95 ± 4.47^a^	3.12 ± 0.73^b^	1.94 ± 0.22^b^	4.22 ± 0.36^b^
TBARS (mg MDA/kg)	0.17 ± 0.03^b^	0.32 ± 0.02^b^	1.73 ± 0.30^a^	1.63 ± 0.42^a^
L*	47.40 ± 4.88^a^	37.13 ± 2.58^b^	39.44 ± 2.85*^ab^*	37.27 ± 0.94^b^
a*	12.38 ± 2.67^a^	18.71 ± 1.21^a^	13.63 ± 1.23^a^	11.15 ± 1.57^a^
b*	13.30 ± 0.98*^ab^*	11.06 ± 1.03^b^	13.70 ± 0.70*^ab^*	14.88 ± 1.18^a^
TSA (log CFU/g)	5.16 ± 0.61^c^	6.76 ± 0.13^b^	7.83 ± 0.52^a^	8.15 ± 0.44^a^
MSA (log CFU/g)	5.17 ± 0.19^c^	6.40 ± 0.40^b^	7.26 ± 0.72*^ab^*	7.83 ± 0.60^a^
MRSA (log CFU/g)	4.97 ± 0.49^c^	6.00 ± 0.59^b^	6.96 ± 0.79^a^	7.18 ± 0.34^a^

Within each row, means with different letters are significantly different (P < 0.05).

Accordingly, other indicators varied with pH and moisture content. TBARS value increased slowly in the early stage (from day 0 to day 7), with no significant differences, and then it increased largely in the middle stage (from day 7 to day 16), with significant differences. The TBARS value of ripened mutton sausage (1.63 mg MDA/kg) formulated in this study was considered to be normal according to the standard ranging from 0.6 to 2.8 mg MDA/kg fermented sausages suggested by Marco et al. (2006). The nitrite ion concentration fell significantly after 7 days of fermentation and then remained quite stable between 1.94 and 4.22 mg/kg in the later period of ripening. The finding of nitrite ion content for mutton sausage in our study was similar to the research conducted by Wang et al. (2013) and Du et al. (2019), with a decreased variation during the whole ripening period. Ripening time affected the lightness (L*), redness (a*), and yellowness (b*). In relation to L* value, a decrease was observed during ripening, since sausages became darker due to weight loss. With respect to a* value, an increase in redness was observed until day 7, followed by a decrease, but with no significant differences. The a* value for mutton sausage increased from 12.38 on day 0 to 18.71 on day 7, suggesting the formation of nitrosomyoglobin with good red color. Although partial nitrite was replaced by nitrate in the formulation of this study, nitrate could be transformed into nitrite by the action of nitrate reductase enzymes present in *Staphylococcus* spp. (Marco et al., 2006) during the curing process. A slight decrease in the b* value at first and then an increase was observed throughout the following process.

Table 1 shows a similar trend of change among the total count of bacteria, presumptive lactic acid bacteria, and *Staphylococci*, which increased during 26 days. Throughout the whole process, the total count of presumptive lactic acid bacteria and *Staphylococci* increased significantly from 0 days to 16 days, reaching about 7 log CFU/g on day 16, followed by an insignificant increase until day 26. The total count of lactic acid bacteria reached a high of 8.62 log CFU/g on day 3 in Xiangxi sausage without the addition of starter cultures (Du et al., 2019), and lactic acid bacteria counts increased after 5 days of drying, reaching 7 log CFU/g in traditional Pitina (Iacumin et al., 2020). Comparatively, it took the total count of presumptive lactic acid bacteria in mutton sausage to reach 7 log CFU/g at least 16 days in our study. The slow increase of lactic acid bacteria and their probably weak acidifying activity resulted in the evolution of the pH level of mutton sausage without a distinct fall. Moreover, Du et al. (2019) showed that the amount of *Staphylococcus* was much lower than lactic acid bacteria in Xiangxi sausage, with 6.5 log CFU/g of *Staphylococcus* after fermentation. Ferreira et al. (2007) also reported that lactic acid bacteria of Portugal’s traditional dry sausages were the dominating microflora with counts above 7.0 log CFU/g comparative to 3.2–4.5 log CFU/g of *Staphylococcus*. In contrast, the *Staphylococci* population of mutton sausage in this study was relatively higher than the presumptive lactic acid bacteria population over the whole process.

### Bacterial diversity of mutton sausage during ripening

The sequencing of the amplicons pool resulted in 1,621,732 paired-end sequence reads overall, with 67,572 sequences per sample. Then, as a result of screening steps, 1,293,824 high-quality sequences overall, with 53,909 sequences per sample, were retained and analyzed. Sequences obtained by high throughput sequencing of amplicons of the bacterial V3–V4 region were analyzed by an OTU (Optional Taxonomic Unit)-based approach, after clustering together sequences with at least 97% similarity.

**FIGURE 1:**
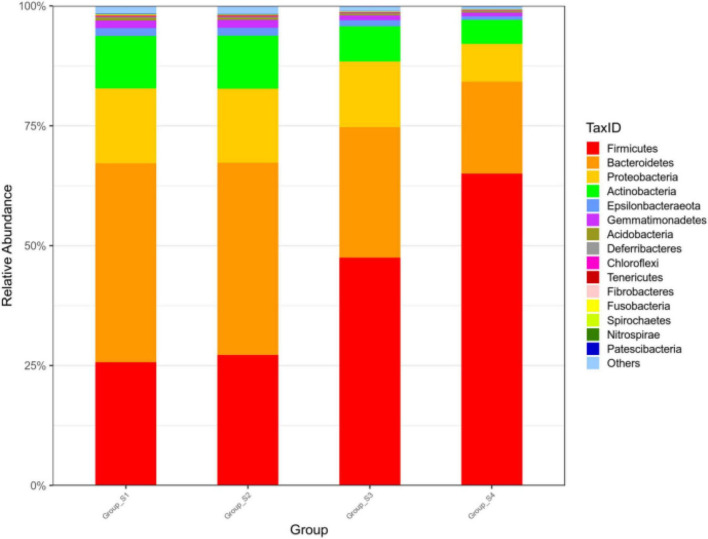
Relative abundance of bacteria at the phylum level of fermented mutton sausage during natural ripening (the 15 most abundant phyla).

As seen in Figure 1, the bacterial relative abundance of the 15 most abundant phyla in the different stages of ripening was analyzed, with percentages of other phyla classified as others. The relative abundance of *Firmicutes* was 25.59% (day 0), 26.99% (day 7), 49.20% (day 16), and 65.83% (day 26), and the relative abundance of *Bacteroidetes* was 41.54% (day 0), 40.34% (day 7), 26.17% (day 16), and 18.67% (day 26). The most abundant phylum in meat on day 0 and day 7 was *Bacteroidetes*, which was replaced by *Firmicutes* increasing to be the most abundant phylum on day 16 and day 26. *Firmicutes* and *Bacteroidetes* became the predominant phyla in all mutton sausage samples and accounted for over 66% of all OTUs, followed in reduced proportion by *Proteobacteria*, *Actinobacteria*, and *Epsilonbacteraeota*.

**FIGURE 2:**
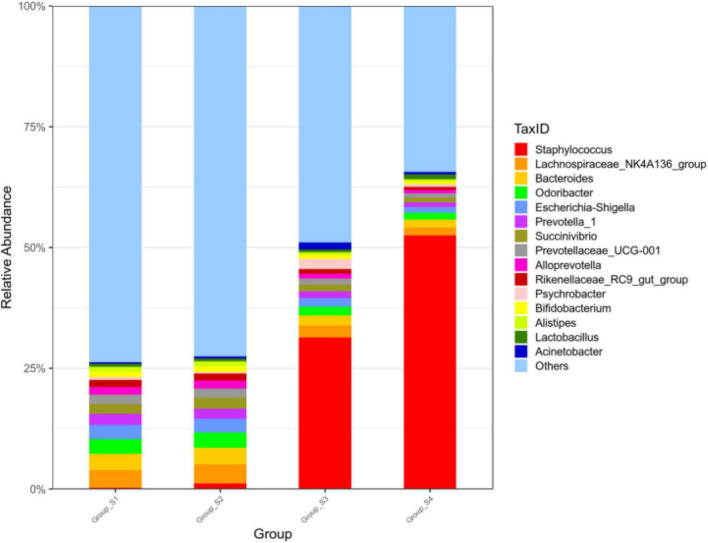
Relative abundance of bacteria at the genus level of fermented mutton sausage during natural ripening (the 15 most abundant genera).

**FIGURE 3:**
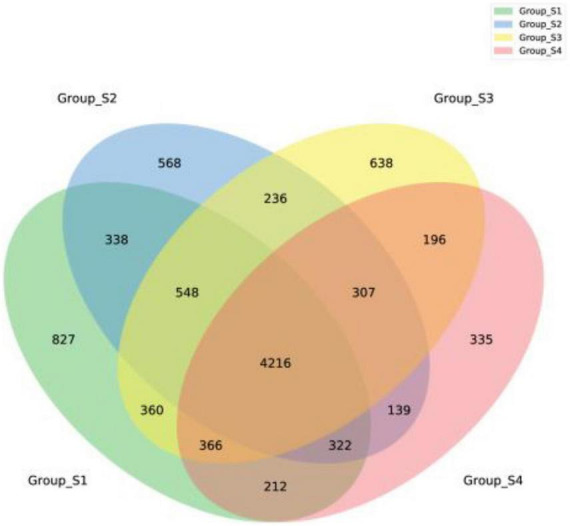
A Venn diagram of fermented mutton sausage during natural ripening based on the OTUs of bacteria.

Figure 2 shows the relative abundance of the 15 most abundant genera in the different ripening stages, with percentages of other genera classified as others. On day 0 and day 7, the *Lachnospiraceae*_NK4A136_group accounted for 3.63 and 3.93%, respectively, followed by a number of taxa identified as *Bacteroides* (3.36 and 3.38%), *Odoribacter* (2.97 and 3.13%), *Prevotella*_1 (2.27 and 2.09%), *Succinivibrio* (1.99 and 2.21%), *Prevotellaceae*_UCG-001 (1.95 and 1.91%), and *Alloprevotella* (1.59 and 1.64%). Most of them are gut microbiota probably from pork intestine casings. After 7 days of ripening, the microbial population was drastically replaced by *Staphylococcu*s. The relative abundance of *Staphylococcus* distinctly increased, reaching abundances of 31.54% on day 16 and abundances of 52.39% until the end of the process, becoming the most dominant genus. Meanwhile, *Lactobacillus* and *Bifidobacterium* maintained a constant relative abundance with low levels throughout the entire process, ranging from 0.58 to 0.99% and from 0.51 to 1.16%, respectively. Apart from *Lactobacillus and Bifidobacterium*, other lactic acid bacteria such as *Leuconostoc*, *Lactococcus*, and *Weissella* were not found within the 30 most abundant genera in all mutton sausage samples. At the same time, the alpha diversity results reported progressive decreases of OTUs and the Chao 1 richness indices and of the Shannon and Simpson diversity indices in Table 2. Moreover, the distribution of OTUs among the different samples was evaluated by a Venn diagram as seen in Figure 3. Totally, 7,189, 6,674, 6,602, and 6,093 OTUs were obtained from days 0, 7, 16, and 26 samples, respectively. Overall, 4,216 OTUs were shared by all samples, which revealed a high-level similarity of bacterial diversity among the samples. Therefore, it could be concluded that the evolution of microbial composition became less rich and diverse.

**TABLE 2:** Alpha diversity indices of fermented mutton sausage during natural ripening.

Samples	Observed OTUs	Goods_ coverage	Chao1	Shannon	Simpson
Day 0	7189	0.99	4164.9	9.95	1
Day 7	6674	0.99	4026.4	9.77	1
Day 16	6602	0.99	3542.3	7.03	0.9
Day 26	6093	0.99	3290.7	5.52	0.76

Chen et al. (2019) found that *Lactobacillus* and *Staphylococcus* in Harbin dry sausages became the dominant genera after 6 days of fermentation, and the abundance of *Lactobacillus* was lower than that of *Staphylococcus*; at the end of the fermentation process, *Staphylococcus* became the most dominant genus, accounting for 42.9%. Hu et al. (2020) demonstrated that *Lactobacillus* and *Staphylococcus* were the predominant genera in traditional dry sausages from different regions in northeast China; *Staphylococcus* was detected at a high relative abundance in Shuangyashan sausage (54.37%) and Heihe sausage (25.23%), with *Lactobacillus* as the sub-major genus in Shuangyashan sausage (2.15%) and Heihe sausage (0.47%); *Lactobacillus* was the major genus in Hegang sausage (37.14%), with the abundance of *Staphylococcus* 8.80%. Wang et al. (2018) showed that *Staphylococcus* was very high in sample SA (a Spanish salami) and SACD (a Chinese dry-cured sausage), accounting for 97.45 and 42.27%, respectively; *Staphylococcus* content (6.78%) was similar to the *Lactobacillus* content (7.93%) in a sample of SACM (a Chinese smoked-cured sausage). Huang et al. (2021) reported that Sichuan traditional fermented sausage samples were predominated by *Lactobacillus* accounting for 53.68% of the total sequence, and *Staphylococcus* was also found for 11.37% of samples. Above all, *Staphylococcus* and lactic acid bacteria corresponding to the phylum *Firmicutes* were recognized as the two main microbial groups that strongly contribute to the fermentation properties in dry fermented sausages by many researchers. The relative abundance of *Staphylococcus* greatly overwhelmed that of *Lactobacillus* for most dry fermented sausages, and *Lactobacillus* became predominant with *Staphylococcus* as a sub-major one for some sausages, and finally, the two genera were close to each other for some other sausages. In our study, *Staphylococcus* composed the vast majority of the bacterial community of dry fermented mutton sausage, and *Lactobacillus* maintaining a relative abundance of below 1% became the sub-major genus; therefore, our fermented mutton sausage was ascribed to the first pattern. The sodium chloride contents of mutton sausages could reach high levels on days 16 and 26, due to the low moisture content of the ripened mutton sausage. Under these high sodium chloride contents, *Staphylococcus* could survive over other bacteria because of its high salt tolerance (Jeong et al., 2014). Therefore, *Staphylococcus* became the most dominant genus from the middle to the end of the ripening stage in the present study.

**FIGURE 4:**
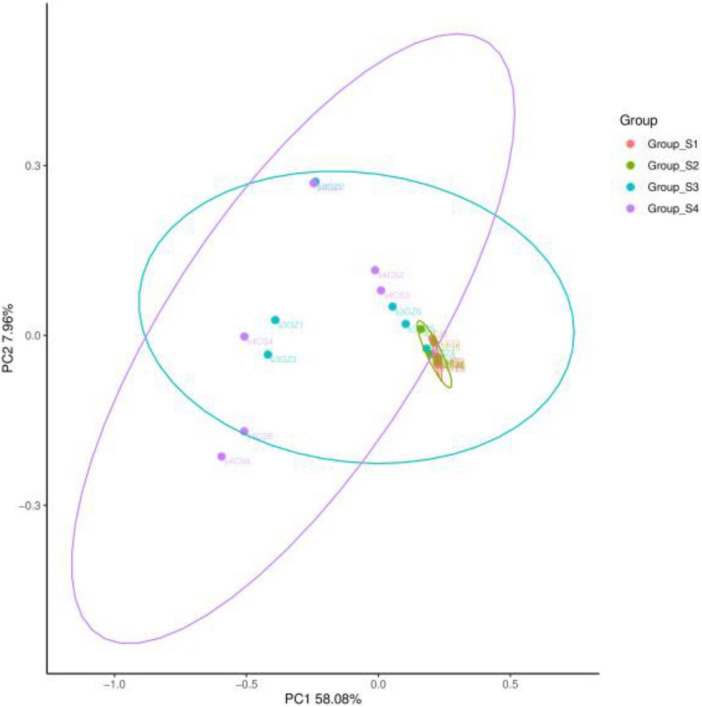
Principal coordinates analysis (PCoA) results of bacterial communities of fermented mutton sausage during natural ripening based on Bray–Curtis distance with 95% confidence level.

The bacterial community was further compared and confirmed using PCoA based on the Bray–Curtis distance matrix (Figure 4). The first and second axes showed values of cumulative percentage variance equal to 58.08 and 7.96%, respectively, and totaled 64.04% variances in OTUs. Totally, day 0 and day 7 samples clustered separately from samples on day 16 and day 26. There was no obvious distinction between day 0 sample and day 7 sample, confirming the structural similarities in the bacterial composition between day 0 and day 7. The distribution of some samples on day 16 at the OTU level overlapped with the former time samples, and some others overlapped with the latter time samples.

**FIGURE 5:**
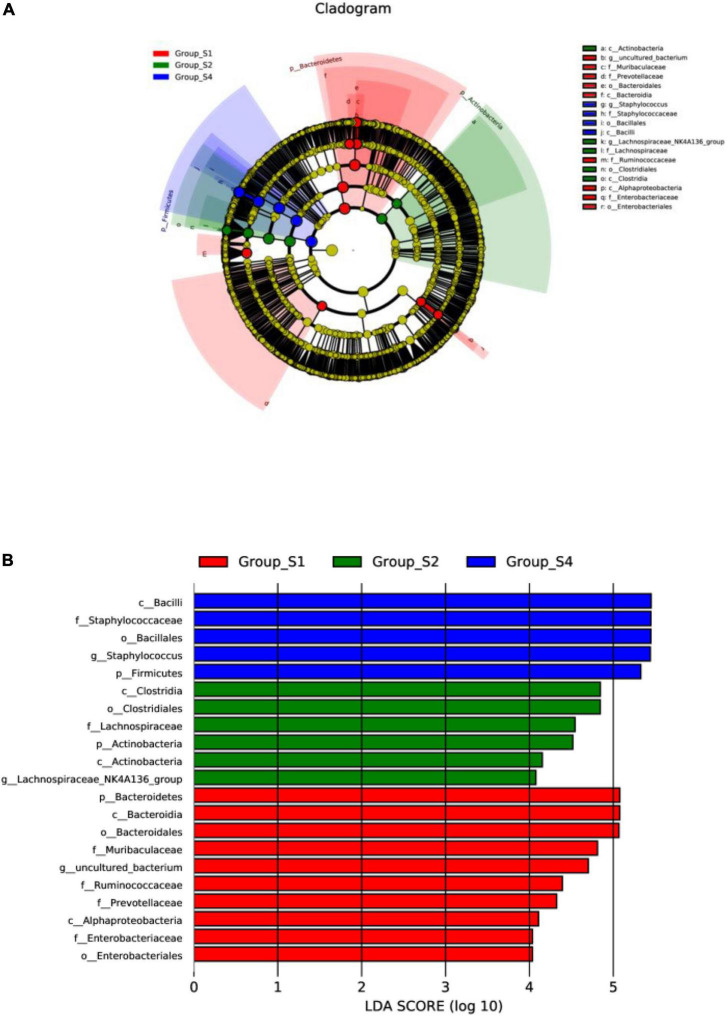
LEfSe highlights consistently differentia bacteria taxa of fermented mutton sausage during natural ripening within four stages. **(A)** Cladogram representation; **(B)** the predominant bacteria of the microbiota, LDA score is over 4. Labels beginning with o_indicate order; f_family; g_genus; s_species.

In addition, linear discriminant analysis coupled with effect size measurements (LEfSe) was performed to obtain the greatest differences in taxa within the different mutton sausages. A total of 438 taxa were found to represent a remarkable difference in their relative abundance (with *P*-value < 0.05, data not shown). Their cladogram representation and the predominant bacteria of the microbiota are shown in Figure 5. Multiple analyses of LEfSe revealed that, during the mutton sausage ripening, 10 differential taxa were observed and the bacterial genus of the uncultured bacterium was clearly distinguishable on day 0. Then, six differential taxa were identified and the most abundant genus of the *Lachnospiraceae*_NK4A136_group was the maker bacteria on day 7. Finally, five differential taxa were determined and the dominant genus of *Staphylococcus* was identified as the characteristic bacteria on day 26. However, none was found to be the characteristic bacteria on day 16.

### Volatile flavors of mutton sausage during ripening

**FIGURE 6:**
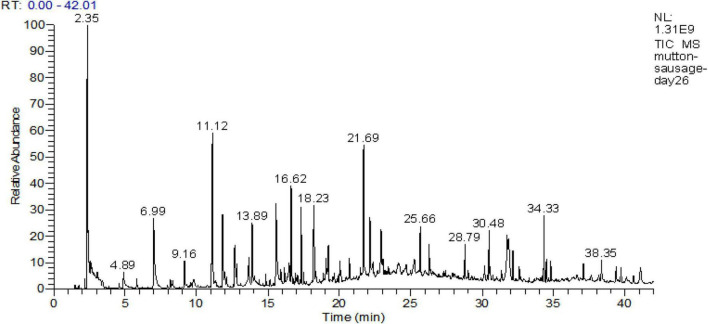
Total ion current chromatogram of fermented mutton sausage on day 26.

The results obtained for the description of the volatile fraction are presented in Table 3. Volatile compounds analysis was performed at all stages of the production process, and the total ion current chromatogram of fermented mutton sausage on day 26 is shown in Figure 6. A total of 44 compounds were detected: aldehydes (14), alcohols (11), ketones (5), carboxylic acids (2), esters (5), lactones (3), pyrazines (2), furans (1), and a miscellanea of one compound. The trend of the concentration of total volatile fraction was to significantly rise from 1,089.76 μg/kg (day 0) to 5,448.77 μg/kg (day 26). So, it was concluded that the majority of flavor compounds were generated at the late ripening of sausage samples.

**TABLE 3:** Contents (μg/kg) of volatile compounds of fermented mutton sausage during natural ripening (means ± standard errors).

Compounds	RI^1^	Threshold^2^ (μg/kg)	Day 0		Day 7		Day 16		Day 26		Aroma descriptor
**Aldehydes (14)**			Content	ROAV	Content	ROAV	Content	ROAV	Content	ROAV	
Hexanal	1126	4.5	28.09 ± 0.09^c^	5.94	13.52 ± 0.28^c^	0.92	234.40 ± 4.49^b^	12.35	498.76 ± 7.07^a^	11.23	Grass, tallow, and fat
*Trans*-2-Octenl	1450	3	17.68 ± 0.39^b^	5.61	n.d.	—	47.79 ± 1.62^b^	3.78	121.15 ± 2.97^a^	4.09	Nut and fat
Decanal	1503	3	53.32 ± 1.15^b^	16.93	58.26 ± 0.66^b^	5.93	151.01 ± 2.62^a^	11.94	150.18 ± 1.38^a^	5.07	Tallow
Benzaldehyde	1547	41.7	326.45 ± 3.44^b^	7.45	129.23 ± 1.76^c^	0.95	315.55 ± 3.76^b^	1.80	639.27 ± 1.83^a^	1.55	Almond and burnt sugar
Dodecyl aldehyde	1715	0.13	13.65 ± 0.99^b^	100.00	18.74 ± 0.58^a^	44.01	27.55 ± 0.28^a^	50.27	n.d.	–	Citrus, tallow, and fat
*Trans*-2-decenal	1676	0.3	17.60 ± 0.25^a^	55.86	n.d.	–	n.d.	–	64.64 ± 0.52^a^	21.84	Cilantro
Non-anal	1571	1.1	n.d.	–	360.35 ± 3.98^b^	100.00	463.76 ± 2.3^b^	100.00	1085.29 ± 9.35^a^	100.00	Fat, citrus, and green
3-methyl-butanal	930	5.8	n.d.	–	n.d.	–	29.56 ± 0.50^a^	1.21	53.54 ± 0.92^a^	0.94	Buttery and nut
Heptanal	1296	2.8	n.d.	–	n.d.	–	67.06 ± 4.23^b^	5.68	203.90 ± 6.00^a^	7.38	Tallow and fat
2-undecenal	1670	5	n.d.	–	n.d.	–	97.75 ± 3.62	4.64	n.d.	–	Tallow and fat
Pentanal	1010	12	n.d.	–	n.d.	–	n.d.	–	50.73 ± 0.87	0.43	Cheesy
Octanal	1222	0.587	n.d.	–	n.d.	–	n.d.	–	339.30 ± 5.04	58.59	Fruity
*Trans-*2-heptenal	1339	0.75	n.d.	–	n.d.	–	n.d.	–	105.25 ± 3.28	14.22	Grass
4-ethylbenzaldehyde	1717	123.23	n.d.	–	n.d.	–	n.d.	–	63.33 ± 1.04	0.05	Fruity and nut
Subtotal			456.79		580.11		1434.77		3375.33		
**Alcohols (11)**											
*Trans*-2-hexen-1-ol	1422	231.9	19.60 ± 0.03	0.08	n.d.	–	n.d.	–	n.d.	–	Fruity
1-octen-3-ol	1579	1.5	36.21 ± 0.70^b^	22.99	30.38 ± 2.22^b^	6.18	155.86 ± 1.09^a^	24.64	243.28 ± 2.68^a^	16.44	Mushroom-like
Heptanol	1646	5.4	11.92 ± 1.46	2.10	n.d.	–	n.d.	–	n.d.	–	Green
1,6-octadien-3-ol, 3,7- dimethyl-,	1553	0.22	9.15 ± 0.39	39.61	n.d.	–	n.d.	–	n.d.	–	Flower, citrus, orange, terpene, waxy, and rose
Undecanol	1830	700	29.13 ± 1.16^a^	0.04	21.52 ± 3.14^a^	0.01	n.d.	–	n.d.	–	Rose
*Trans*-2-nonen-1-ol	1616	209	336.68 ± 3.15	1.53	n.d.	–	n.d.	–	n.d.	–	Violet like
Non-anol	1638	45.5	15.94 ± 0.27^a^	0.33	10.85 ± 0.46^a^	0.07	n.d.	–	n.d.	–	Rose
3-methylbutanol	1217	4	28.37 ± 1.21	6.75	n.d.	–	n.d.	–	n.d.	–	Brandy like, apple
Pentanol	1241	150.2	n.d.	–	n.d.	–	n.d.	–	34.04 ± 1.67	0.02	Fruity, alcoholic, green, balsamic, and woody
Octanol	1520	125.8	n.d.	–	n.d.	–	116.08 ± 3.49	0.22	n.d.	–	Chemical, metal, and burnt
Benzyl alcohol	1900	2546.21	n.d.	–	n.d.	–	n.d.	–	74.36 ± 2.12	0.003	Flower
Subtotal			487.00		57.33		271.93		351.68		
**Ketones (5)**											
6,10-dimethyl-5,9-undecadien-2-one	1935	60	29.71 ± 0.93^c^	0.47	36.57 ± 0.39^c^	0.19	76.46 ± 0.90^b^	0.30	110.94 ± 4.3^a^	0.19	Spice
4-hydroxy-4-methyl-2-pentanone	1804	–	23.42 ± 2.19^b^	–	25.69 ± 3.50^b^	–	190.61 ± 4.16^a^	–	44.01 ± 1.25^b^	–	–
2-Non-anone	1419	41	18.37 ± 0.27	0.43	n.d.	–	n.d.	–	n.d.	–	Rose and tea
Acetoin	1340	14	n.d.	–	10.02 ± 0.04	0.22	n.d.	–	n.d.	–	Cream
Benzophenone	2975	–	n.d.	–	n.d.	–	21.55 ± 0.43^b^	–	104.64 ± 0.23^a^	–	Rose
Subtotal			71.51		72.29		288.62		259.59		
**Esters (5)**											
Methyl myristate	2219	–	n.d.	–	10.07 ± 0.45	–	n.d.	–	n.d.	–	Honey
Tributyrin	2457	–	n.d.	–	37.39 ± 0.59^a^	–	45.78 ± 0.62^a^	–	n.d.	–	Oil, fat, and tallow
Amyl butyrate	1478	210	n.d.	–	n.d.	–	35.03 ± 2.03	0.04	n.d.	–	Sweety
Butyl butyrate	1492	400	n.d.	–	n.d.	–	n.d.	–	53.50 ± 2.39	0.01	Fruity
Hexyl formate	1642	–	18.06 ± 0.02^a^	–	n.d.	–	26.19 ± 2.02^a^	–	n.d.	–	Fruity
Subtotal			18.06		47.47		107.00		53.50		
**Lactones (3)**											
γ-dodecalactone	2332	0.43	36.52 ± 1.86^b^	80.88	23.22 ± 2.06^b^	16.48	55.57 ± 4.12^b^	30.66	121.12 ± 4.05^a^	28.55	Fruity
δ-tetradecalactone	2150	29000	n.d.	–	n.d.	–	19.07 ± 1.12	0.00002	n.d.	–	–
2(3H)-Furanone, dihydro-5-(2Z)-2-octen-1-yl-	2401	–	n.d.	–	n.d.	–	17.37 ± 0.66	–	n.d.	–	–
Subtotal			36.52		23.22		92.02		121.12		
**Acids (2)**											
4-methylvaleric acid	2471	810	n.d.	–	14.30 ± 0.17	0.01	n.d.	–	n.d.	–	Acid
2-methylbutyric acid	1704	5800	n.d.	–	n.d.	–	34.27 ± 2.23	0.0014	n.d.	–	Roquefort
Subtotal			0.00		14.30		34.27		0.00		
**Pyrazines (2)**											
Tetramethylpyrazine	1439	2525.02	n.d.	–	n.d.	–	144.98 ± 6.12^b^	0.01	1006.46 ± 36.47^a^	0.04	Tallow and fat
2,3-dimethylpyrazine	1385	800	n.d.	–	n.d.	–	n.d.	–	172.80 ± 4.80	0.02	Meaty and cream
Subtotal			0.00		0.00		144.98		1179.26		
**Furans (1)**											
2-pentylfuran	1252	5.8	11.17 ± 0.38^c^	1.83	12.30 ± 0.20^c^	0.65	45.70 ± 4.67^b^	1.87	108.31 ± 4.89^a^	1.89	Grass and fruity
Subtotal			11.17		12.30		45.70		108.31		
**Others (1)**											
2-methoxyphenol	1902	1.6	8.70 ± 0.51	5.18	n.d.	–	n.d.	–	n.d.	–	Meaty
Subtotal			8.70		9.23		0.00		0.00		

Within each row, means with different letters are significantly different (P < 0.05). n.d. not detected.

^1^Retention index calculated for TG-WAXMS B capillary column (30 m × 0.25 mm × 0.25 μm).

^2^Odor threshold value referred to Compilations of odor threshold values in air, water, and other media (second enlarged and revised edition). van Gemert (2011): Science Press.

The aldehydes detected in all stages were not only in a high total amount but also contained a large number, including 12 aliphatic aldehydes (7 kinds of alkanals, 4 kinds of 2-alkenals, and 1 kind of methyl-branched aldehyde) and 2 aromatic aldehydes. During the production process, a distinct increase in aldehydes concentration was noted. The minimal concentration of aldehydes was observed after curing (456.79 μg/kg on day 0), while the highest one was detected at the end of processing (3,375.33 μg/kg on day 26). Out of 14 different kinds of aldehydes, nonanal, benzaldehyde, hexanal, and decanal were present in a high percentage: the percentage of nonanal accounted for 0% (day 0), 62.12% (day 7), 32.32% (day 16), and 32.15% (day 26) of the total volatile fraction, and the percentage of benzaldehyde was 71.46% (day 0), 22.27% (day 7), 22.02% (day 16), and 18.94% (day 26) of the total volatile fraction for four sample points. Hexanal and decanal were present throughout the whole ripening in the percentage of 2 ∼ 17% and 4 ∼ 12%, respectively. Non-anal, hexanal, decanal, heptanal dodecyl aldehyde, and benzaldehyde were considered key volatile compounds of fermented mutton sausage, attributing to their presence (at least two ripening time points) and ROAVs above 1. According to the report of Domínguez et al. (2019), pentanal and hexanal were linoleic acid oxidative compounds, and heptanal, octanal, nonanal, and decanal were oleic acid oxidative compounds. These aldehydes were all detected during the production, especially present in very high concentrations at the end of the ripening of mutton sausage. It could be speculated that the components of Jianzhou Big-Eared mutton might be rich in oleic acid and linoleic acid. Oleic acid was demonstrated to be the prominent fatty acid of mutton fat (Nizar et al., 2013) and also was higher in ewe as well as lamb musculus biceps femoris meat (Junkuszew et al., 2020). On the contrary, branched-chain amino acids (leucine, isoleucine, and valine) are converted into methyl-branched aldehydes with malty flavors, depending on which amino acid is catabolized (Ardö, 2006). In our study, 3-methylbutanal derived from leucine appeared from day 16 and increased to day 26. It was considered a key volatile compound of fermented mutton sausage owing to its presence on both day 16 and day 26 and ROAV at approximately around 1.

Alcohols and lactones showed an irregular trend: a higher concentration of alcohols was observed on day 0, reaching a value of 487.00 μg/kg and a percentage of 44.7% of the total volatile fraction, while the alcohols’ lower level was detected on day 7. The lowest concentration of lactones was similarly found on day 7 in line with alcohols, whereas lactones reached the highest concentration of 121.12 μg/kg on day 26. Pentanol and benzyl alcohol were not detected during the 16-day period but appeared at the endpoint of processing on day 26. An opposite trend was observed for *trans*-2-nonen-1-ol, which was detected in high concentrations (336.68 μg/kg) only on day 0. Out of the three lactones identified, γ-dodecalactone was the most predominant one, characterized by high ROAV at four time points and considered a key volatile of fermented mutton sausage.

Ketones and esters showed a similar trend: They were present in lower concentrations in the early production stages (from day 0 to day 7), which then increased up to the maximum on day 16, comprising 11.9% for ketones and 3.8% for esters of the total volatile compounds. After that, a decrease was detected during the next ripening period. 6,10-dimethyl-5,9-undecadien-2-one and 4-hydroxy-4-methyl-2-pentanone were detected along the whole process of ripening, whereas they were excluded from key volatiles of fermented mutton sausage, owing to ROAV being lower than 1 for 6,10-dimethyl-5,9-undecadien-2-one and the absence of aroma characteristics for 4-hydroxy-4-methyl-2-pentanone. In addition, 2-non-anone and acetoin were only detected at one time point during the process and also at a relatively low concentration.

Notably, both 1-octen-3-ol and 2-pentylfuran were observed with high concentrations over the course of ripening, which were especially higher in the late ripening period, and were considered to be key volatile compounds of fermented mutton sausage due to their ROAVs being above 1. 1-octen-3-ol exhibits a strong mushroom flavor even in high dilution (Wnuk et al., 1983). 1-octen-3-ol is a characteristic fragrance compound produced by *Aspergillus*, such as *Aspergillus luchuensis* (Kataoka et al., 2020), *Aspergillus flavus* (Miyamoto et al., 2014), and so on. Moreover, 2-pentylfuran was commonly known and definitely confirmed to be produced primarily by fungal genera in the report of Elmassry et al. (2020). Therefore, it could be inferred that certain amounts of fungi might be existing in our mutton sausage. This point should be confirmed by following experiments about the mold diversity of naturally fermented mutton sausage.

**FIGURE 7:**
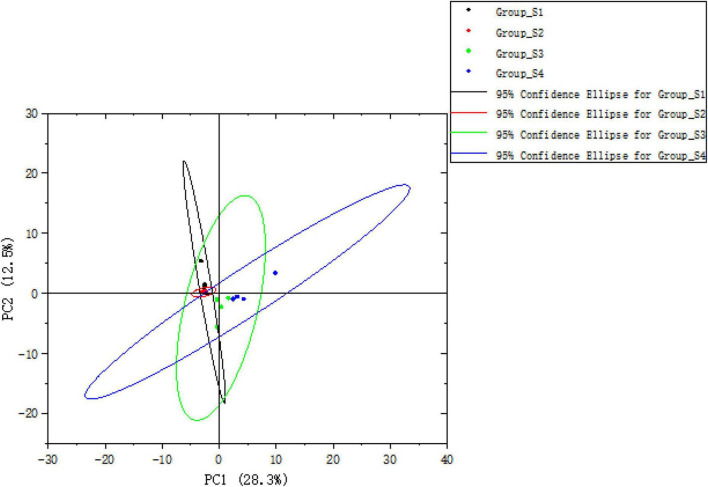
The PCA score plot of total volatile compounds of fermented mutton sausage on days 0, 7, 16, and 26.

The PCA was used to analyze the variation and similarity of the volatile profiles among mutton sausages from different stages. In total, 44 volatile compounds detected were used for the PCA score plot, as seen in Figure 7. The PCA score plot showed that 40.8% of the variation among samples could be explained by two components (PC1 28.3% and PC2 12.5%). The results revealed that the day 0 sample and the day 7 sample were clustered together, suggesting that they had a similar volatile profile. The day 16 sample and day 26 sample were nearly separated, indicating that they had certain differences in volatile profiles.

### Correlation between bacteria and volatiles

**FIGURE 8:**
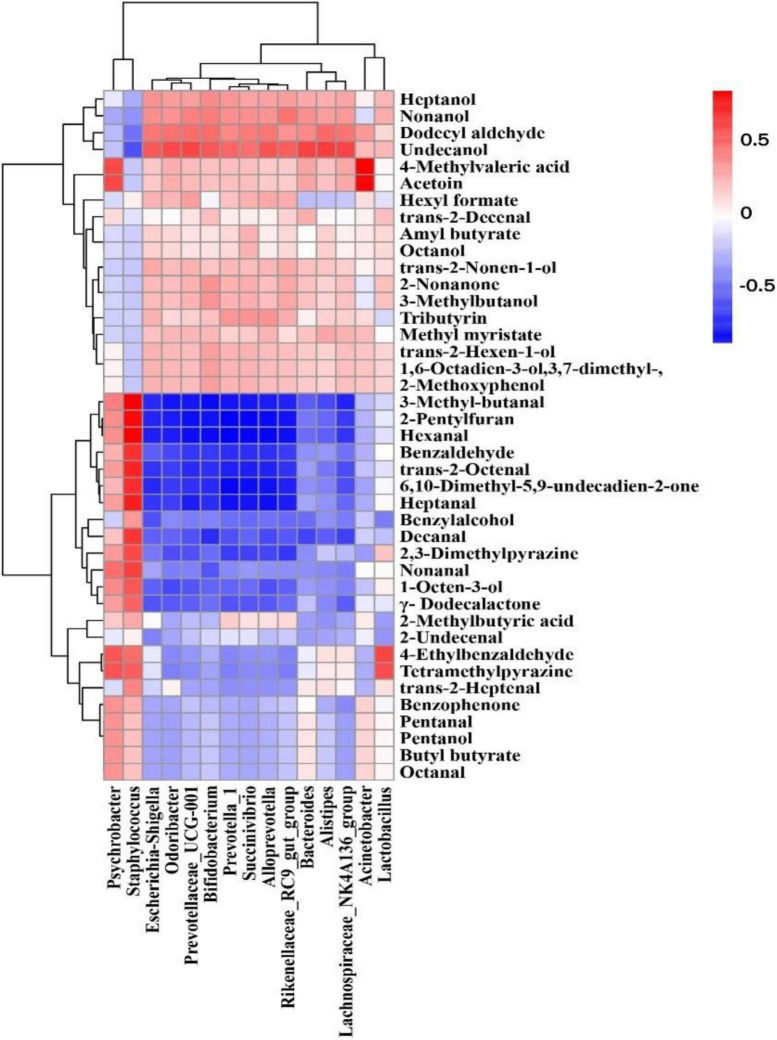
A heatmap of the Spearman rank correlation between genera with relative abundance in the top 15 and the volatile properties in fermented mutton sausage during natural ripening. The intensity of the colors represents the degree of correlation between the genera and volatile compounds, where the blue color represents a negative degree of correlation and red a positive correlation.

A Spearman rank correlation analysis was conducted to reveal the relationship between bacterial composition (dominant genera with relative abundance top 15) and volatile compounds (except for 4-hydroxy-4-methyl-2-pentanone, δ-tetradecalactone, and 2(3H)-furanone, dihydro-5-(2Z)-2-octen-1-yl- without aroma characteristics) of different mutton sausage samples using a clustering heat map (Figure 8). The results showed that different microorganisms contributed differently to the volatile flavors.

There are strong positive correlations between *Staphylococcus* and *Psychrobacter* and the production of most aldehydes (3-methyl-butanal, hexanal, benzaldehyde, *trans*-2-octenal, heptanal, decanal, nonanal), γ-dodecalactone, 1-octen-3-ol, 2-pentylfuran, benzophenone, 6,10-dimethyl-5,9-undecadien-2-one, 2,3-dimethylpyrazine, and tetramethylpyrazine. *Bifidobacterium, Prevotellaceae*_UCG-001, *Succinivibrio*, *Escherichia-Shigella*, *Prevotella*_1, *Rikenellaceae_*RC9_gut_group, *Bacteroides*, *Alloprevotella*, *Odoribacter*, *Lachnospiraceae*_NK4A136_group, *Acinetobacter*, *Alistipes, and Lactobacillus* were associated with the production of most alcohols and esters, namely heptanol, nonanol, undecanol, octanol, *trans*-2-nonen-1-ol, 3-methylbutanol, *trans*-2-hexen-1-ol, 1,6-octadien-3-ol,3,7- dimethyl-, 2-methoxyphenol, hexyl formate, amyl butyrate, tributyrin, methyl myristate, 4-methylvaleric acid, and acetoin. In addition, *Lactobacillus* was positively correlated with 4-ethylbenzaldehyde and tetramethylpyrazine. From the above results, it could be deduced that the predominant bacteria had a significant contribution to the formation of volatile compounds in naturally fermented mutton sausage.

### Sensory analysis

The sensory attributes are shown in Table 4. Appearance and total acceptability increased with increased ripening time, whereas day 26 had a lower score compared to day 16 based on the increase of darkness and hardness due to much higher water loss. Color (the intensity of red) reached the maximum on day 16 and was not significantly different from day 7, which was consistent with the results obtained with the colorimeter. Flavor increased with increased ripening time, and no significant differences were observed between day 16 and day 26. This was in accordance with the results of volatile detection, with the concentration of total volatile fraction rising at the late ripening time.

**TABLE 4:** Sensory quality (means ± standard error) of fermented mutton sausage during natural ripening.

Parameters	Day 0	Day 7	Day 16	Day 26
Appearance	1.75 ± 0.25^c^	5.00 ± 0.82^b^	7.00 ± 0.41^a^	5.25 ± 0.63*^ab^*
Color	2.00 ± 0.41^b^	6.50 ± 0.64^a^	6.75 ± 0.48^a^	5.75 ± 0.48^a^
Flavor	1.75 ± 0.25^b^	5.25 ± 0.75^a^	6.50 + 0.29^a^	6.25 ± 1.18^a^
Overall acceptability	1.75 ± 0.48^c^	5.00 ± 0.82^b^	7.25 ± 0.48^a^	5.50 ± 0.50*^ab^*

Means within each row with different superscript letters are significantly different (P < 0.05).

## Conclusion

Physicochemical properties of mutton sausage during the natural ripening process were recorded. *Firmicutes* and *Bacteroidetes* accounted for over 66% of all OTUs throughout the whole process, with *Lachnospiraceae*_NK4A136_group and *Staphylococcus* as the predominant genus at the early and later ripening periods, respectively. The evolution of microbial composition became less rich and diverse. The uncultured bacterium, *Lachnospiraceae*_NK4A136_group, and *Staphylococcus* were marker bacteria on days 0, 7, and 26, respectively. However, none was found to be the characteristic bacterium for day 16. The structure similarities in the bacterial composition were observed between day 0 and day 7 through PCoA analysis, separately from day 16 and day 26. On the contrary, samples of day 0 and day 7 had a similar volatile profile and samples of day 16 and day 26 had certain differences in volatile profiles in terms of PCA analysis. The strain distribution seemed to influence the volatile profile of mutton sausage throughout processing. The concentration of total volatile fraction significantly increased and the majority of volatile compounds were generated during late ripening. Non-anal, hexanal, decanal, heptanal, dodecyl aldehyde, benzaldehyde, 3-methylbutanal, γ-dodecalactone, 2-pentylfuran, and 1-octen-3-ol were identified as key volatile compounds of the dry fermented mutton sausage. Based on Spearman’s correlation analysis, *Staphylococcus* as well as *Psychrobacter* were positively correlated with the production of the key volatile compounds, and other bacteria such as *Lachnospiraceae*_NK4A136_group, *Bacteroides*, *Lactobacillus*, *Prevotella*_1, *Odoribacter*, and so on were associated with the production of most alcohols and esters.

## Data availability statement

The data presented in this study are deposited in Sequence Read Archive, submission number: SUB11888604 and the data has been released, accession number: PRJNA865401. This data can be found here: https://dataview.ncbi.nlm.nih.gov/?archive=bioproject/PRJNA865401.

## Author contributions

JC: writing – review and editing. YN: investigation. JW: methodology. ZY: software. ZC: formal analysis. XD: formal analysis. CW: project administration. YW: supervision. YL: funding acquisition. All authors contributed to the article and approved the submitted version.

## Footnotes

1
http://www.flavornet.org/flavornet.html

## References

[B1] Ardö Y. (2006). Flavour formation by amino acid catabolism. *Biotechnol. Adv.* 24 238–242. 10.1016/j.biotechadv.2005.11.005 16406465

[B2] Arief I. I. Wulandari Z. Aditia E. Baihaqi M. Noraimah Hendrawan. (2014). Physicochemical and microbiological properties of fermented lamb sausages using probiotic *Lactobacillus plantarum* IIA-2C12 as starter culture. *Procedia Environ. Sci.* 20 352–356. 10.1016/j.proenv.2014.03.044

[B3] Bratcher C. Dawkins N. Solaiman S. Kerth C. Bartlett J. (2011). Texture and acceptability of goat meat frankfurters processed with 3 different sources of fat. *J. Anim. Sci.* 89 1429–1433. 10.2527/jas.2010-3398 21183711

[B4] Chen J. X. Hu Y. Y. Wen R. X. Liu Q. Chen Q. Kong B. H. (2019). Effect of NaCl substitutes on the physical, microbial and sensory characteristics of Harbin dry sausage. *Meat Sci.* 156 205–213. 10.1016/j.meatsci.2019.05.035 31202095

[B5] Domínguez R. Pateiro M. Gagaoua M. Barba F. J. Zhang W. G. Lorenzo J. M. (2019). A comprehensive review on lipid oxidation in meat and meat products. *Antioxidants* 8:429. 10.3390/antiox8100429 31557858 PMC6827023

[B6] Du S. Cheng H. Ma J. K. Li Z. J. Wang C. H. Wang Y. L. (2019). Effect of starter culture on microbiological, physiochemical and nutrition quality of Xiangxi sausage. *J. Food Sci. Technol.* 56 811–823. 10.1007/s13197-018-3541-z 30906039 PMC6400778

[B7] Elmassry M. M. Farag M. A. Preissner R. Gohlke B.-O. Piechulla B. Lemfack M. C. (2020). Sixty-one volatiles have phylogenetic signals across bacterial domain and fungal kingdom. *Front. Microbiol.* 11:2380. 10.3389/fmicb.2020.557253 33101231 PMC7554305

[B8] Ferreira V. Barbosa J. Silva J. Vendeiro S. Mota A. Silva F. (2007). Chemical and microbiological characterisation of “Salpicão de Vinhais” and “Chouriça de Vinhais”: Traditional dry sausages produced in the North of Portugal. *Food Microbiol.* 24 618–623. 10.1016/j.fm.2006.12.007 17418313

[B9] Ferrocino I. Bellio A. Giordano M. Macori G. Romano A. Rantsiou K. (2018). Shotgun metagenomics and volatilome profile of the microbiota of fermented sausages. *Appl. Environ. Microbiol.* 84:e02120–17. 10.1128/stocktickerAEM.02120-17 29196291 PMC5772244

[B10] Flores M. Piornos J. A. (2021). Fermented meat sausages and the challenge of their plant-based alternatives: A comparative review on aroma-related aspects. *Meat Sci.* 182:108636. 10.1016/j.meatsci.2021.108636 34314926

[B11] Hu Y. Y. Zhang L. Liu Q. Wang Y. Chen Q. Kong B. H. (2020). The potential correlation between bacterial diversity and the characteristic volatile flavour of traditional dry sausages from Northeast China. *Food Microbiol.* 91:103505. 10.1016/j.fm.2020.103505 32539975

[B12] Huang Z. C. Shen Y. Huang X. Q. Qiao M. W. He R. K. Song L. J. (2021). Microbial diversity of representative traditional fermented sausages in different regions of China. *J. Appl. Microbiol.* 130 133–141. 10.1111/jam.14648 32219941

[B13] Iacumin L. Osualdini M. Bovolenta S. Boscolo D. Chiesa L. Panseri S. (2020). Microbial, chemico-physical and volatile aromatic compounds characterization of Pitina PGI, a peculiar sausage-like product of North East Italy. *Meat Sci.* 163:108081. 10.1016/j.meatsci.2020.108081 32062526

[B14] Jeong D.-W. Han S. Lee J.-H. (2014). Safety and technological characterization of *Staphylococcus equorum* isolates from jeotgal, a Korean high-salt-fermented seafood, for starter development. *Int. J. Food Microbiol.* 188 108–115. 10.1016/j.ijfoodmicro.2014.07.022 25106039

[B15] Junkuszew A. Nazar P. Milerski M. Margetin M. Brodzki P. Bazewicz K. (2020). Chemical composition and fatty acid content in lamb and adult sheep meat. *Arch. Anim. Breed.* 63 261–268. 10.5194/aab-63-261-2020 32775611 PMC7405649

[B16] Kataoka R. Watanabe T. Yano S. Mizutani O. Yamada O. Kasumi T. (2020). *Aspergillus luchuensis* fatty acid oxygenase ppoC is necessary for 1-octen-3-ol biosynthesis in rice koji. *J. Biosci. Bioeng.* 129 192–198. 10.1016/j.jbiosc.2019.08.010 31585859

[B17] Marco A. Navarro J. L. Flores M. (2006). The influence of nitrite and nitrate on microbial, chemical and sensory parameters of slow dry fermented sausage. *Meat Sci.* 73 660–673. 10.1016/j.meatsci.2006.03.011 22062567

[B18] Miyamoto K. Murakami T. Kakumyan P. Keller N. P. Matsui K. (2014). Formation of 1-octen-3-ol from *Aspergillus* flavus conidia is accelerated after disruption of cells independently of Ppo oxygenases, and is not a main cause of inhibition of germination. *PeerJ* 2:e395. 10.7717/peerj.395 24883255 PMC4034645

[B19] Nizar N. N. A. Marikkar J. M. N. Hashim D. M. (2013). Differentiation of lard, chicken fat, beef fat and mutton fat by GCMS and EA-IRMS techniques. *J. Oleo Sci.* 62 459–464. 10.5650/jos.62.459 23823911

[B20] O’Keefe S. F. Wang H. (2006). Effects of peanut skin extract on quality and storage stability of beef products. *Meat Sci.* 73 278–286. 10.1016/j.meatsci.2005.12.001 22062299

[B21] Połka J. Rebecchi A. Pisacane V. Morelli L. Puglisi E. (2015). Bacterial diversity in typical Italian salami at different ripening stages as revealed by high-throughput sequencing of 16S rRNA amplicons. *Food Microbiol.* 46 342–356. 10.1016/j.fm.2014.08.023 25475305

[B22] Quijada N. M. De Filippis F. Sanz J. J. García-Fernández M. de C. Rodríguez-Lázaro D. (2017). High-throughput sequencing analysis reveals different *Lactobacillus* populations that dominate during â Chorizo de LeÃłnâ cured meat making. *Food Microbiol.* 70 94–102. 10.1016/j.fm.2017.09.009 29173645

[B23] Ravyts F. Vuyst L. D. Leroy F. (2012). Bacterial diversity and functionalities in food fermentations. *Eng. Life Sci.* 12 356–367. 10.1002/elsc.201100119

[B24] Stajić S. Perunoviæ M. Stanišiæ N. Žujoviæ M. Živkoviæ D. (2013). Sucuk (Turkish-style dry-fermented sausage) quality as an influence of recipe formulation and inoculation of starter cultures. *J. Food Process. Preserv.* 37 870–880. 10.1111/j.1745-4549.2012.00709.x

[B25] Talon R. Leroy S. (2011). Diversity and safety hazards of bacteria involved in meat fermentations. *Meat Sci.* 89 303–309. 10.1016/j.meatsci.2011.04.029 21620574

[B26] Talon R. Leroy S. Lebert I. (2007). Microbial ecosystems of traditional fermented meat products: The importance of indigenous starters. *Meat Sci.* 77 55–62. 10.1016/j.meatsci.2007.04.023 22061396

[B27] Teixeira A. Silva S. Guedes C. Rodrigues S. (2020). Sheep and goat meat processed products quality: A review. *Foods* 9:960. 10.3390/foods9070960 32698535 PMC7404805

[B28] van Gemert L. J. (2011). *Compilations of odour threshold values in air, water and other media (Second enlarged and revised edition)*. Utrecht: Oliemans Punter & Partners BV.

[B29] Wang D. B. Zhao L. H. Su R. N. Jin Y. (2019). Effects of different starter culture combinations on microbial counts and physico-chemical properties in dry fermented mutton sausages. *Food Sci. Nutr.* 7 1957–1968. 10.1002/fsn3.989 31289643 PMC6593374

[B30] Wang X. H. Zhang Y. L. Ren H. Y. Zhan Y. (2018). Comparison of bacterial diversity profiles and microbial safety assessment of salami, Chinese dry-cured sausage and Chinese smoked-cured sausage by high-throughput sequencing. *LWT Food Sci. Technol.* 90 108–115. 10.1016/j.lwt.2017.12.011

[B31] Wang X. Ren H. Liu D. Zhu W. Wang W. (2013). Effects of inoculating *Lactobacillus sakei* starter cultures on the microbiological quality and nitrite depletion of Chinese fermented sausages. *Food Control* 32 591–596. 10.1016/j.foodcont.2013.01.050

[B32] Wnuk S. Kinastowski S. Kaminski E. (1983). Synthesis and analysis of l-octen-3-ol, the main flavour component of mushrooms. *Food Nahrung* 27 479–486. 10.1002/food.19830270523 6684212

[B33] Zhang H. Huang D. Pu D. Zhang Y. Chen H. Ren F. Z. (2020). Multivariate relationships among sensory attributes and volatile components in commercial dry porcini mushrooms (*Boletus edulis*). *Food Res. Int.* 133:109112. 10.1016/j.foodres.2020.109112 32466923

[B34] Zhao L. Jin Y. Ma C. Song H. Li H. Wang Z. (2011). Physico-chemical characteristics and free fatty acid composition of dry fermented mutton sausages as affected by the use of various combinations of starter cultures and spices. *Meat Sci.* 88 761–766. 10.1016/j.meatsci.2011.03.010 21458169

